# A pilot study of the relative number of circulating tumor cells and leukocytes containing actin-binding proteins in head and neck cancer patients

**DOI:** 10.7555/JBR.36.20220182

**Published:** 2022-11-10

**Authors:** Gelena Kakurina, Marina Stakheeva, Elena Sereda, Evgenia Sidenko, Olga Cheremisina, Evgeny Choinzonov, Irina Kondakova

**Affiliations:** 1 Cancer Research Institute, Tomsk National Research Medical Center, Russian Academy of Sciences, Tomsk 634009, Russia; 2 Department of Biochemistry and Molecular Biology, Faculty of Medicine and Biology, Siberian State Medical University, Tomsk 634050, Russia

**Keywords:** head and neck squamous cell carcinoma, metastasis, circulating tumor cells, actin-binding proteins, adenylyl cyclase-associated protein 1

## Abstract

Circulating tumor cells (CTCs) play an important role in tumor metastases, which is positively correlated with an increased risk of death. Actin-binding proteins, including cofilin (CFL1), profilin 1 (PFN1), and adenylate cyclase-associated protein 1 (CAP1), are thought to be involved in tumor cell motility and metastasis, specifically in head and neck squamous cell carcinoma (HNSCC). However, currently, there are no published studies on CFL1, PFN1, and CAP1 in CTCs and leukocytes in HNSCC patients. We assessed serum levels of CFL1, PFN1, and CAP1 and the number of CTCs and leukocytes containing these proteins in blood from 31 HNSCC patients (T1–4N0–2M0). The analysis used flow cytometry and an enzyme-linked immunosorbent assay kit. We found that CAP1^+^ CTCs and CAP1^+^ leukocyte subpopulations were prevalent in these HNSCC patient samples, while the prevalence rates of CFL1^+^ and PFN1^+^ CTCs were relatively low. Patients with stage T2–4N1–2M0 had CFL1^+^ and PFN1^+^ CTCs with an elevated PFN1 serum level, compared with the T1–3N0M0 group. In summary, the PFN1 serum level and the relative number of PFN1^+^CD326^+^ CTCs could be valuable prognostic markers for HNSCC metastases. The current study is the first to obtain data regarding the contents of actin-binding proteins (ABPs) in CTCs, and leukocytes in blood from HNSCC patients. This is also the first to assess the relationship between the number of CTCs subgroups and disease characteristics.

## Introduction

The reorganization of the actin cytoskeleton associated with the epithelial-mesenchymal transition (EMT) is regulated by various actin-binding proteins (ABPs), which plays a key role in tumor cell migration^[1–3]^. Many studies on the role of the actin cytoskeleton in tumor progression have focused on ABPs, specifically cofilin-1 (CFL1) and profilin-1 (PFN1)^[4]^. The participation of these proteins in the reorganization of actin filaments has also been verified by numerous investigators, and has therefore been entered into the BioGRID and STRING databases (https://thebiogrid.org and https://cn.string-db.org, respectively). CFL1 is involved in the disassembly of F-actin, PFN1 is a monomer- binding protein, and these proteins function in tandem, while ABPs regulate actin cytoskeletal dynamics (https://thebiogrid.org). At present, the aforementioned ABPs are being studied as potential markers of cancer aggressiveness^[4]^ (https://www.proteinatlas.org). There is also evidence that adenylyl cyclase-associated protein 1 (CAP1) is involved in regulating functional activity of CFL1^[5]^. Indeed, *in vivo* experiments of yeast have shown that CAP1 is also a physiological partner of profilin during actin reorganization^[6]^. However, there is no study on the simultaneous participation of these three proteins in cancer development or progression.

Despite several reports evidencing an opposing effect of CFL1 overexpression in tumor tissues, many authors maintain that this protein has more potential as a prognostic marker for tumor recurrence^[4,7]^. However, specific molecular mechanisms requiring CFL1 involvement in the formation of malignant phenotypes of tumor cells are yet to be described^[7]^. In addition, CFL1 activation is thought to play a key role in the chemotactic response of T-lymphocytes^[8]^. Therefore, the targeted manipulation of immune cells within tumor microenvironment may solve the problem of chemoresistance, at least for some solid tumors. This may also open up new areas of research and help develop novel approaches for cancer immunotherapeutics.

CFL1 has been shown to function both independently and in conjunction with CAP1^[5]^. CAP1 is involved in the regulation of actin filament remodeling by binding to both forms of actin (*i.e.*, G-actin and F-actin), which determines its role in cancer progression^[9–12]^. There is also evidence of the involvement of CAP1 in signaling pathways^[9,11–12]^ and caveolin-dependent endocytosis, in particular, in the internalization of low-density lipoprotein receptor (LDLR) and low density lipoprotein receptor-related protein (LRP)^[13–14]^. Recently, there have also been a number of studies suggesting that CFL1 and CAP1 work in conjunction to influence the aggressiveness of tumor development^[10,12,14–15]^.

The involvement of PFN1 in the processes of tumor progression is still being studied^[4,16–18]^. However, the dual role of PFN1 in oncogenesis has been confirmed by several investigators through numerous basic studies. For example, it has been reported that reduced PFN1 expression is correlated with an increased metastatic activity in breast cancer in female nude mice^[17]^. However, an increased expression of* PFN1* mRNA, together with the increased expression of other ABPs (*e.g.*, CFL1, enabled/vasodilator-stimulated phosphoprotein, and actin capping protein), is also associated with a poor prognosis in clear-cell renal cell carcinoma (CCRCC)^[18]^. The involvement of PFN1 in carcinogenesis is likely to be determined not only by the histotype and localization of the tumor, but also by its relationship with other proteins. Therefore, further studies are required.

Tumor expansion is associated with the appearance of circulating tumor cells (CTCs) in bloodstream, which is associated with a poorer prognoses in cancer in various localizations^[1,19–20]^. The determination of CTCs for screening, monitoring of responses to anticancer treatments, and predicting outcomes of breast, prostate and colorectal cancers has been evidenced^[20]^. For additional CTC characterization with an epithelial phenotype, the use of cytokeratins and EpCAM is recommended. However, it has been pointed out that there is a limitation to this analytical method. That is, CTCs that have undergone EMT are not detectable because they no longer express EpCAM or CK^[21–22]^. Therefore, given the important role of ABPs in cellular motility and the lack of data regarding the CTC architecture cytoskeleton, it is important to study the levels of CFL1, PFN1, and CAP1 in CTCs and systemic leukocytes in cancer patients.

The most suitable model for us to study was head and neck squamous cell carcinoma (HNSCC). HNSCC is a highly aggressive tumor characterized by late-stage diagnosis and poor outcomes. One of the causes of death in patients with HNSCC is the development of lymph node metastases. CTCs are implicated in the development and progression of metastases. It has been previously shown that CTC levels may be important for HNSCC prognostics^[23]^; however, further studies of CTC subpopulations are needed. The current study aimed to analyze the number of CTCs and leukocytes containing CFL1, PFN1, and CAP1 in the peripheral blood of patients with HNSCC. The number of these cells was also assessed to the main clinical and pathological parameters, such as sex, tumor localization, and lymph node metastasis. In addition, the correlation between the levels of these proteins in blood serum and the number of CTCs and leukocytes containing the above-mentioned proteins were assessed.

## Subjects and methods

### Patients

The current study included 31 patients with histologically verified stage T1–4N0–2M0 HNSCC, who were treated at the Department of Head and Neck Cancer of Cancer Research Institute (Tomsk, Russia) between 2020 and 2021. The median age was 56.5 years (range: 41 to 68 years). Patient characteristics are provided in ***Tables 1*** and ***2***. All patients were also tested for the presence of coronavirus disease COVID-19. Blood work included the counts of white blood cells (WBCs), and the percentages of lymphocytes and monocytes in WBCs.

**Table 1:** Clinical characteristics of patients with head and neck squamous cell carcinoma

Characteristics	*n* (%)
Sex	
Male	26 (83.9)
Female	5 (16.1)
Age (years)	
≤52	4 (12.9)
>52	27 (87.1)
Tumor localization	
Oral cavity	14 (45.2)
Laryngopharynx	17 (54.8)
T stage	
T1–2N0–1M0	12 (38.7)
T3–4N0–2M0	19 (61.3)
N stage (lymphogenous metastasis)	
T1–4N0M0	13 (41.9)
T1–4N1–2M0	18 (58.1)
Pathological differentiation	
Low	4 (12.9)
Middle	21 (67.7)
High	4 (12.9)
Undifferentiated	2 (6.5)
COVID-19	
Positive	3 (9.7)
Negative	28 (90.3)
T1–4N0M0: patients with head and neck squamous cell carcinoma having no lymph node metastases; T1–4N1–2M0: patients with head and neck squamous cell carcinoma with histologically confirmed lymph node metastases. COVID-19: coronavirus disease 2019.	

**Table 2:** The histological characteristics of the epithelial tissue, sex, tumor localization, and the presence of metastases in lymph nodes of the neck of HNSCC patients

Characteristics	T1–4N0M0 [*n*(%)]	T1–4N1–2M0 [*n*(%)]	Totals [*n*(%)]
Male, oral cancer			
Keratinizing	5 (16.1)	2 (6.4)	7 (22.6)
Non-keratinizing	2 (6.4)	0 (0.0)	2 (6.4)
Male, laryngeal cancer			
Keratinizing	1 (3.2)	5 (16.1)	6 (19.4)
Non-keratinizing	5 (16.1)	2 (6.4)	7 ( 22.6)
Female, oral cancer			
Keratinizing	0 (0.0)	5 (16.1)	5 (16.1)
Non-keratinizing	0 (0.0)	4 (12.9)	4 (12.9)
Female, laryngeal cancer			
Keratinizing	0 (0.0)	0 (0.0)	0 (0.0)
Non-keratinizing	0 (0.0)	0 (0.0)	0 (0.0)
General group (%)	13 (41.9)	18 (58.1)	31 (100.0)
T1–4N0M0: patients with head and neck squamous cell carcinoma having no lymph node metastases; T1–4N1–2M0: patients with head and neck squamous cell carcinoma with histologically confirmed lymph node metastases. HNSCC: head and neck squamous cell carcinoma.			

The study was carried out in accordance with the Declaration of Helsinki, "Ethical principles for conducting scientific medical research involving humans" (amended in the 2000 version). The study was approved by the local ethics committee of the Cancer Research Institute of Tomsk National Research Medical Center (Approval No. 14-07\20). Informed consent for the study was obtained from each patient.

### Enzyme-linked immunosorbent assay

Blood serum was obtained according to an approved protocol, and stored at −80 ℃. Levels of PFN1, CFL1, and CAP1 in the blood serum of HNSCC patients were analyzed using a Multiskan FC microplate reader (Thermo Scientific, Shanghai, China). Enzyme-linked immunosorbent assay (ELISA) kits for the detection of CAP 1, PFN1, CFL1 were purchased from Cloud-Clone Corp. (China; Cat. Nos. SEB349Hu, SEC233Hu, and SEB559Hu). Optical densities of samples and standards were measured at the wavelength specified in the kit manufacturer's instructions. Protein concentration was calculated using Magellan software (Tecan, Switzerland).

### Flow cytometric analysis

For the analysis, peripheral blood (8 mL) from each patient was collected into BD Vacutainer Plus vacuum tubes with EDTA (BD, USA) and used immediately according to the approved protocol. The number of leukocytes and CTCs in whole blood samples was assessed by flow cytometry on a BD FACSCanto Ⅱ flow cytometer (BD). Blood samples were divided into 100 μL aliquots for further analysis. To prevent nonspecific binding of monoclonal and polyclonal antibodies to Fc receptors on the cell surface, samples were incubated with Human TruStain FcX reagent (BioLegend Inc., US) before staining the surface molecules according to the instructions.

At the first stage, membrane markers CD45, CD326, and CAP1 were stained using the corresponding antibodies (***Table 3***). Lysing solution (BD) was then added to remove erythrocytes from the sample. In the second stage, after preliminary permeabilization of the cell membrane, intracellular ASBs (*i.e.*, CAP1, PFN1, and CFL1) were stained using appropriate antibodies (***Table 3***). The analysis was carried out using a set of staining transcription factors (Transcription Factor Buffer Set, BD, USA) according to the associated instructions. Thresholds were set on fluorescence minus one (FMO) and isotype controls. A compensation matrix was generated automatically using the BD FACS Diva Software after data acquisition.

**Table 3:** Antibodies used for flow cytometry

Antibodies	Protein	Catalog No.	Manufacturer
Alexa Fluor 700 Mouse Anti-human CD45	CD45	560566	BD, San Jose, California, USA
PerCP-Cy 5.5 Mouse Anti-Human EpCAM	Human EpCAM	ABIN650759	BD, San Jose, California, USA
APC-Linked Anti-Cofilin 1 (CFL1) Polyclonal antibody	Human cofilin 1		LSBio, Seattle, Washington, USA
Alexa Fluor 647-Linked Anti-Profilin 1 (PFN1) Polyclonal antibody, [EPR6304]	Human profilin 1	ab124904	AssayPro, St. Charles, MO, USA
Anti-CAP1 antibody [EPR8339(B)]	Adenylyl cyclase-associated protein 1	ab155079	Abcam, Cambridge, UK
Goat Anti-Rabbit IgG H&L (Alexa Fluor 488)	–	ab150077	Abcam, Cambridge, UK

The gating strategy included steps for separating blood cells into CD45^+^ cells (leukocytes) and CD45^−^ cells. Gates of CAP1^+^, PFL1^+^, and CFL1^+^ cells were sequentially isolated from the gate of CD45^+^ leukocytes, and their numbers were presented as a percentage of the pool of leukocytes. CD326^+^ (EpCAM^+^) cells were isolated from the gate of CD45^−^ cells, accepting these cells as CTCs. From this gate, the cell populations were also sequentially isolated as a percentage of the CTCs pool. The results are presented as a percentage of CD45^+^ and EpCAM^+^ cells expressing the investigated markers of ABPs (*i.e.*, CAP1, PFL1, and CFL1).

### Statistical analysis

Data were processed statistically using the Statistical Program for Social Science (SPSS), version 20.0 software (SPSS Inc., USA) for Windows and the Statistica (version 6.0) software package (StatSoft Inc., USA). Normal distribution was checked using the Shapiro-Wilk's test. Results of data with non-Gaussian distribution are described using median and interquartile range (IQR). Differences between groups were assessed using non-parametric Kruskal-Wallis' and Mann-Whitney's tests. Spearman's rank correlation coefficient (*r*) was used to measure the strength and direction of the association between the ranked variables. For all types of analysis, *P-*values of less than 0.05 were considered statistically significant. The effectiveness of the selected markers was assessed using the receiver operating characteristic (ROC) curve, with the area under the ROC curve (AUC) and with 95% confidence interval (CI).

In addition, one-year metastasis-free survival in HNSCC patients was assessed. Metastasis-free survival was defined as the time interval from the end of the combined modality treatment to the time of the patient's last visit to an oncologist or until an initial metastasis. The follow-up period was between three and 12 months. The one-year metastasis-free survival rate in HNSCC patients was 46%, with a median survival time of five months. When assessing the association between the content of ABPs and the one-year metastasis-free survival rates, the generalized log-rank test and the *χ*2 test were used to compare among multiple groups. Survival curves were constructed using the Kaplan-Meier method.

## Results

### Quantitative analysis of actin-binding proteins: PFN1, CFL1, and CAP1

In our samples of patients with stage T1–4N0–2M0 HNSCC, the median levels of CAP1, CFL1, and PFN1 in the serum were 0.10 ng/mL (IQR 0.07–0.15 ng/mL), 0.47 ng/mL (IQR 0.37–0.82 ng/mL), and 0.32 ng/mL (IQR 0.25–0.42 ng/mL), respectively. Therefore, the CFL1 level in the serum of HNSCC patients was higher than the CAP1 and PFN1 levels (*P*=0.001).

**Figure 1:**
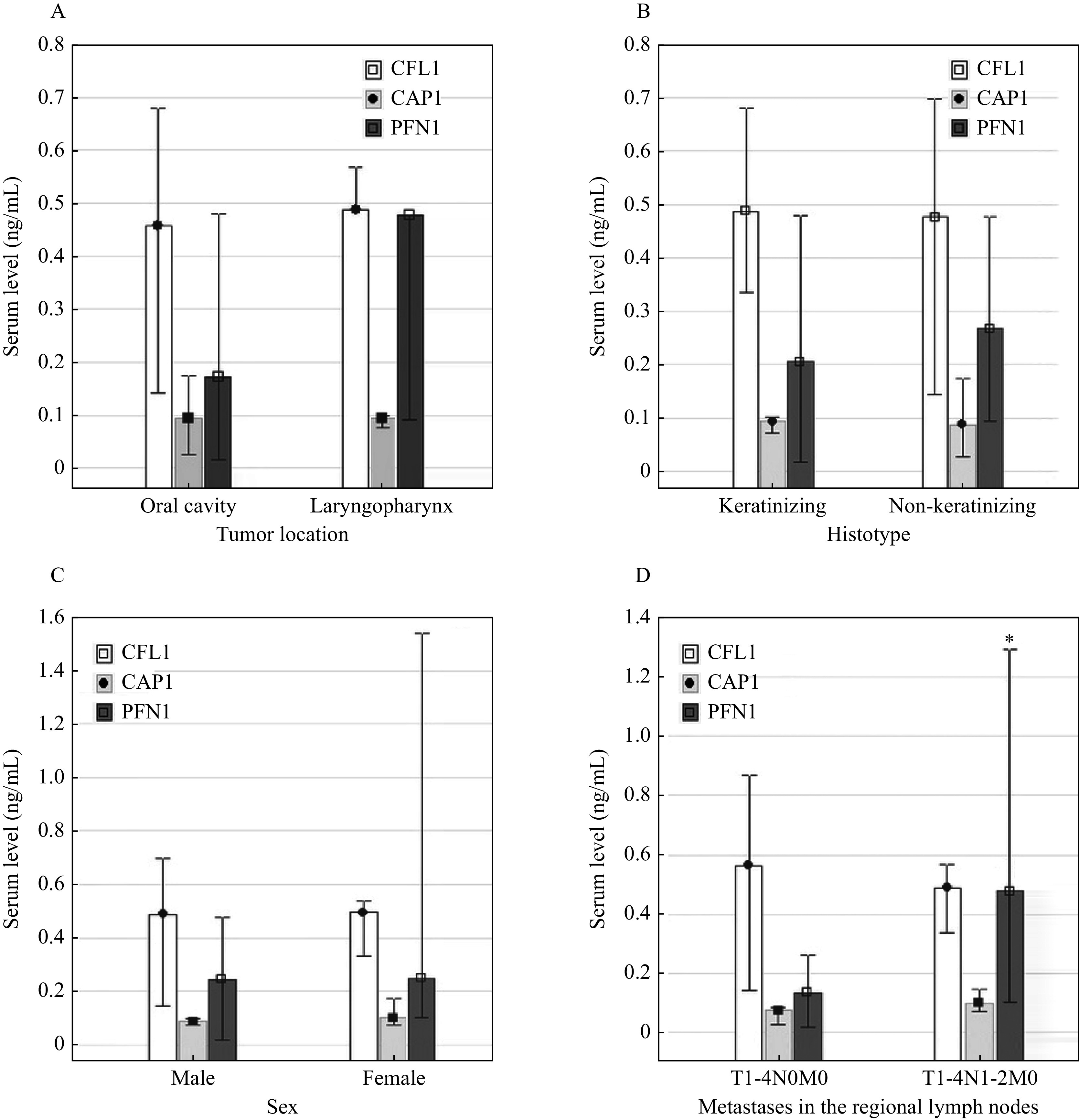
The levels of ABPs in the blood serum of HNSCC patients in relation to the main clinical pathological characteristics.

Tumor location (***Fig. 1A***), cytological characteristics of epithelial cells (***Fig. 1B***), and sex of the patients (***Fig. 1C***) did not have a statistically significant dependence on the level of the studied ABPs. In HNSCC patients with histologically confirmed lymph node metastases (T1–4N1–2M0), the median level of PFN1 in the blood serum was almost two times higher than in patients without regional metastases (T1–4N0M0) (***Fig. 1D***). Therefore, PFN1 represents a potential predictor for metastases in patients with HNSCC.

### Correlations between peripheral leukocytes, lymphocytes and monocytes and the main clinical and morphological parameters

Possible correlations between agranulocytes and the number of ABP-expressing cells of the total leukocyte pool, the total WBC count, as well as the relative lymphocyte and monocyte counts were assessed. The percentage of lymphocytes in WBCs was reduced by 35% in HNSCC patients with histologically confirmed lymph node metastases (T2–4N1–2M0), compared with those patients without lymph node metastases (T1–3N0M0), 20.00% (16.05%, 23.81%) and 31.00% (29.71%, 38.00%), respectively (*P*≤0.05). The relative number of leukocytes was lower in women than in men, 16.05% (13.50%, 23.25%) and 26.75 (20.85%, 34.50%), respectively (*P*=0.06). In HNSCC patients with non-keratinized epithelium, the relative number of leukocytes was higher than that in HNSCC patients without keratinization of the epithelium, 30.71% (23.80%, 38.00%) and 18.00% (16.10%, 21.70%), respectively (*P*=0.08). These results did not highlight significant differences.

### The number of CTCs in the peripheral blood of patients appears to be related to metastasis

The relative number of CTCs in the peripheral blood of HNSCC patients was determined using the flow cytometry method. In 31 of HNSCC patients, the relative number of CTCs to the total number of cells (per 5000 blood cells) in the peripheral blood was 0.04% (IQR 0–0.07%). In HNSCC patients without lymph node metastases (T1–3N0M0), CTCs were not detected, while the median values for T3N1–2M0 and T4N1–2M0 HNSCC were 0.01% (IQR 0–0.08%) and 0.04% (IQR 0.03%–0.07%), respectively.

### There was a difference between the levels of CD45^+^ leukocytes and CD45^−^CD326^+ ^CTCs containing actin-binding proteins in the peripheral blood

Relative quantitative analysis of cells containing ABPs (*i.e.*, CFL1, CAP1, and PFN1) from the total pool of CD45^+^ leukocytes and the population of CD45^−^CD326^+^ CTCs was carried out using flow cytometry. The results showed that the total pool of CD45^+^ leukocytes mainly consisted of CFL1^+^ and CAP1^+^ subpopulations: 18.32% (IQR 8.01%–38.60%) and 38.80% (IQR 8.09%–56.09%), respectively, and the CD45^−^CD326^+^ CTC population was most represented by CAP1^+^ (87.74% [IQR 76.53%–99.30%]). The analysis using the Wilcoxon signed-rank test was conducted with a Bonferroni correction, resulting in a significance level of* P*<0.01.

A comparative assessment of cell pools containing the studied proteins in relation to the main clinical and pathological parameters was carried out. Analysis of the number of ABP-expressing subpopulations in the total pool of CD45^+^ leukocytes did not reveal significant differences in the clinical and pathological parameters between the subgroups (***Table 4***).

**Table 4:** Analysis of the correlations between the CD45^+ ^leukocytes subpopulations containing ABPs in peripheral blood and the main clinical and pathological parameters of HNSCC patients

Characteristics	CFL1			CAP1			PFN1	
	CFL1^+^ leukocytes (%)	*P*-value		CAP1^+^ leukocytes (%)	*P*-value		PFN1^+^ leukocytes (%)	*P*-value
Sex		0.52			0.83			0.78
Male	16.50 (9.10–39.60)			40.03 (18.30–54.00)			8.10 (4.06–10.76)	
Female	11.35 (9.10–25.45)			43.30 (4.18–82.55)			5.60 (3.91–16.22)	
Tumor localization		0.85			0.75			0.65
Oral cancer	11.81 (9.09–39.07)			51.10 (4.20–67.59)			5.61 (4.09–12.29)	
Laryngeal cancer	19.76 (13.31–20.65)			36.65 (18.30–42.40)			10.54 (4.30–10.82)	
Histotype		0.17			0.52			0.13
Keratinizing	19.81 (11.57–41.41)			35.73 (6.42–54.86)			10.46 (4.12–25.32)	
Non-keratinizing	11.21 (7.92–20.67)			48.20 (37.60–55.71)			4.21 (3.73–9.58)	
Metastasis		0.28			0.41			0.68
T1–3N0M0	11.56 (2.80–55.31)			54.02 (1.87–55.69)			4.29 (3.08–15.04)	
T2–4N1–2M0	20.22 (7.90–41.37)			36.67 (2.01–84.85)			8.81 (3.69–38.31)	
T1–3N0M0: no metastasis; T2–4N1–2M0: 1 or 2 lymph nodes are involved. Results were presented as percentage of CD45^+^ leukocytes expressing the above ABPs. Differences between groups were assessed using nonparametric Kruskal-Wallis and Mann-Whitney *U* test. Data are presented as median (interquartile range). ABPs: actin-binding proteins; HNSCC: head and neck squamous cell carcinoma; CFL1: cofilin-1; CAP1: adenylyl cyclase-associated protein 1; PFN1: profilin-1.								

Analysis of ABP-containing CTC subpopulations (CD45^−^CD326^+^) in the peripheral blood of patients with HNSCC in relation to metastasis revealed a significant difference in the number of subpopulations of CFL1^+^CD326^+^ CTCs and PFN1^+^CD326^+^ CTCs (***Table 5***). It should be noted that in HNSCC patients with no histologically confirmed lymph node metastases (T1–3N0M0), the CD45^−^CD326^+^ CTCs population did not contain subpopulations expressing CFL1 and PFN1 proteins.

**Table 5:** Analysis of the correlations between the CD45^−^CD326^+^ CTCs subpopulations containing ABPs in peripheral blood and the main clinical and morphological parameters of HNSCC patients

Characteristics	CFL1			CAP1			PFN1	
	CFL1^+^ CTCs (%)	*P*-value		CAP1^+^ CTCs (%)	*P*-value		PFN1^+^ CTCs (%)	*P*-value
Sex		0.62			0.73			0.58
Male	0.00 (0.00–1.00)			78.40 (71.00–100.00)			0.05 (0.00–1.55)	
Female	0.00 (0.00–25.00)			95.80 (56.91–99.35)			0.45 (0.21–1.35)	
Tumor localization		0.41			0.40			0.45
Oral cancer	0.00 (0.00–0.53)			86.95 (63.91–99.35)			0.05 (0.06–0.45)	
Laryngeal cancer	0.00 (0.00–3.95)			78.68 (78.13–100.00)			1.54 (0.00–1.65)	
Histotype		0.08			**0.01**			0.29
Keratinizing	0.00 (0.00–3.95)			74.00 (53.80–81.00)			0.40 (0.00–2.20)	
Non-keratinizing	0.00 (0.00–0.00)			100.00 (92.90–100.00)			0.00 (0.00–0.50)	
Metastasis		**0.04**			0.38			**<0.01**
T1–3N0M0	0.00 (0.00–0.00)			100.00 (53.80–100.00)			0.00 (0.00–0.00)	
T2–4N1–2M0	0.50 (0.00–50.00)			79.84 (20.90–100.00)			1.02 (0.00–16.70)	
T1–3N0M0: no metastasis; T2–4N1–2M0: 1 or 2 lymph nodes are involved. Results were presented as percentage of CD45^−^CD326^+^ CTCs expressing the above ABPs. Data are presented as median (interquartile range). Differences between groups were assessed using nonparametric Kruskal-Wallis and Mann-Whitney *U* test. Bold font indicates *P*-value<0.05. CTC: circulating tumor cells; ABPs: actin-binding proteins; HNSCC: head and neck squamous cell carcinoma; CFL1: cofilin-1; CAP1: adenylyl cyclase-associated protein 1; PFN1: profilin-1.								

We also analyzed CTC subpopulations containing ABPs in patients with keratinizing and non-keratinizing HNSCC. In patients with keratinizing squamous cell carcinoma, the CTC subpopulation expressing CAP1 was lower by 26%, compared with that in patients without keratinizing squamous cell carcinoma (*P*=0.01) (***Table 5***). It should be noted that in the peripheral blood of patients with squamous cell carcinoma of the oral cavity and larynx, the CTC subpopulation containing CAP1 was most abundantly represented. The content of CAP1 in CTCs has not been studied previously. We found that the serum levels of CAP1 were much lower than those of CFL1 and PFN1.

Molecular characterization of CTCs based on proteins involved in cytoskeletal remodeling, in particular ABPs, will likely allow the creation of more accurate prognostic tools. The high aggressiveness of HNSCC and the increased scientific interest in CTCs as a possible prognostic tool for cancer of this localization provides a need for a comprehensive study of the molecular characteristics of these cells and the relevance of our study.

### Correlations between pools of CD326^+^ CTCs and CD45^+^ leukocytes expressing CFL1, PFN1, and CAP1, serum levels of ABPs and pathological parameters

**Figure 2:**
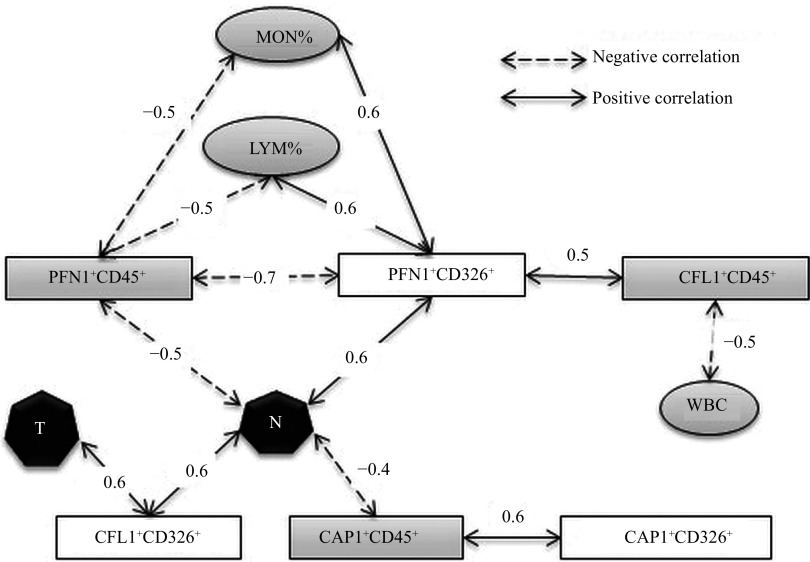
Correlation between subpopulations of CD326^+^ CTCs and CD45^+^ leukocytes expressing ABPs, serum levels of ABPs and the main clinical and pathological parameters (criteria N and T) in HNSCC patients.

Numerous correlations of different directions and average strength were identified between the studied indicators (***Fig. 2***). The number of PFN1^+^ and CFL1^+^CD326^+^ CTCs was positively correlated with lymph node metastases. The tumor size (T1–4) was positively correlated with the number of CFL1^+^CD326^+^ CTCs, and the type of epithelium was correlated with the number of CAP1^+^CD326^+^ CTCs. A positive correlation between CAP1^+^CD326^+^ CTCs and CAP1^+^CD45^+^ leukocytes was also observed. The number of PFN1^+^CD326^+^ CTCs was positively correlated with the content of lymphocytes and monocytes, but was negatively correlated with the number of PFN1^+^CD45^+^ leukocytes. Additionally, both subpopulations isolated from the blood containing PFN1 were correlated negatively with each other. The level of CFL1^+^CD45^+^ leukocytes was negatively correlated with the total number of leukocytes. The results obtained indirectly suggested the functional relationship between these proteins and HNSCC progression.

The possibility of using the serum level of ABPs, the number of CD45^+^ leukocytes and CD326^+^ CTCs containing these ABPs to predict lymph node metastasis in patients with HNSCC was evaluated by the ROC analysis. Significant predictive values were found for serum levels of CAP1 and PFN1 as well as for the number of PFN1^+^CD326^+^ CTCs (***Table 6***).

**Table 6:** Area under the ROC for candidate markers of lymph node metastasis in HNSCC patients

Candidate markers	AUC (95% CI)	*P*-value	Sensitivity (%)	Specificity (%)
CFL1				
CD45^+^ leukocytes	0.62 (0.37–0.32)	0.32	62	70
CD326^+^ CTCs	0.69 (0.48–0.12)	0.11	40	98
Blood serum	0.47 (0.17–0.80)	0.80	85	52
CAP1				
CD45^+^ leukocytes	0.52 (0.25–0.90)	0.90	34	30
CD326^+^ CTCs	0.43 (0.17–0.55)	0.55	50	50
Blood serum	0.82 (0.61–0.01)	**0.01**	84	70
PFN1				
CD45^+^ leukocytes	0.62 (0.39–0.32)	0.32	64	50
CD326^+^ CTCs	0.92 (0.80–1.00)	**0.00**	85	96
Blood serum	0.78 (0.58–0.03)	**0.03**	77	70
Bold font indicates *P*-value<0.05. ROC: receiver operating characteristic; HNSCC: head and neck squamous cell carcinoma; CI: confidence interval; AUC: area under the ROC curve; CTC: circulating tumor cells; CFL1: cofilin-1; CAP1: adenylyl cyclase-associated protein 1; PFN1: profilin-1.				

Therefore, the assessment of the number of PFN1^+^CD326^+^ CTCs in the peripheral blood of HNSCC patients can determine the presence of lymph node metastases with a sensitivity of 85% and a specificity of 96%.

### The metastasis-free survival in patients with head and neck squamous cancer cells depending on the profilin-1 serum levels

When analyzing the survival rate, the calculation was carried out in the first year after surgery, taking into account the patients who were lost to follow-up and who died from concomitant non-cancer diseases. The metastasis-free survival rates were calculated in months from the operative therapy until the time of the patient's last visit to the oncologist or the identification of the outcome (cancer progression). The follow-up period ranged between two and 12 months.

Within a 12-month follow-up, disease progression (lymph node metastases) occurred in 54.8% (17 patients out of 31) of the patients. The median values of the corresponding parameters were chosen as reference levels dividing the content of CAP1, CFL1, and PFN1 into high and low levels (***Table 7***).

**Table 7:** The one-year metastasis-free survival rates depending on ABPs in the serum of patients with HNSCC

ABPs in serum	Number of patients (*n*)	Cumulative proportion of survivors (%)	*P*-value
СAP1 (ng/mL)			0.12
>0.097	20	57.3	
≤0.097	11	15.7	
CFL1 (ng/mL)			0.38
>0.67	14	32.4	
≤0.67	17	76.5	
PFN1 (ng/mL)			0.07
>0.32	17	11.0	
≤0.32	14	64.9	
*P*-values were determined by log-rank test. ABPs: actin-binding proteins; HNSCC: head and neck squamous cell carcinoma; CAP1: adenylyl cyclase-associated protein 1; CFL1: cofilin-1; PFN1: profilin-1.			

**Figure 3:**
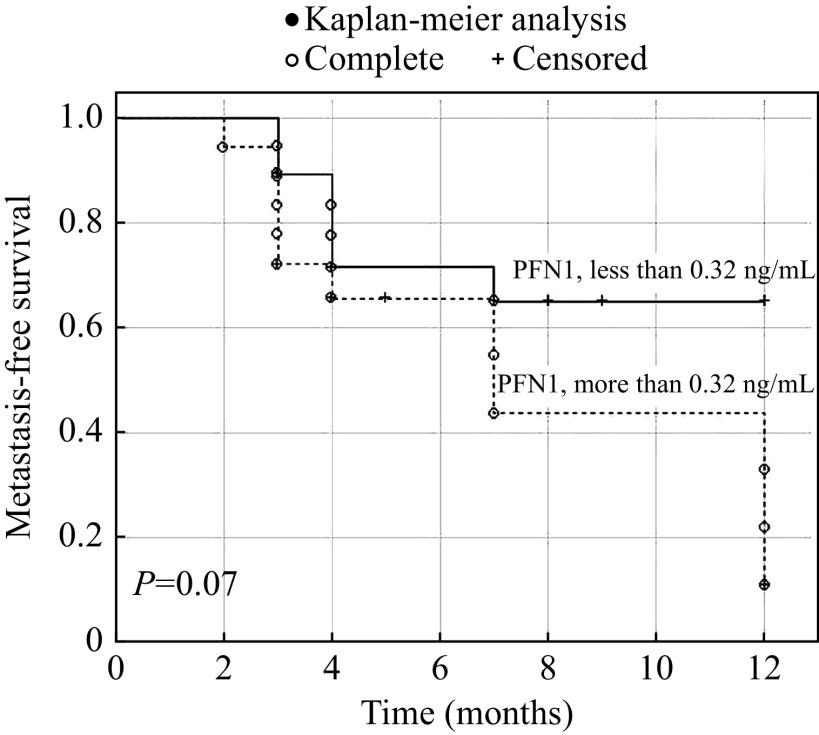
Kaplan-Meier analysis of the one-year metastasis-free survival in HNSCC patients according to PFN1 serum levels.

At a 12-month follow-up, disease progression (occurrence of lymph node metastases) occurred in 64.7% (11 patients out of 17) of HNSCC patients with a high serum level of PFN1 (more than 0.32 ng/mL) and only in 42.8 % (6 patients out of 14) of the patients with a non-significantly low serum level of PFN1 (less than or equal to 0.32 ng/mL) (*P*=0.07) (***Fig. 3***).

## Discussion

The presence of CTCs is associated with a poorer prognosis in various types of tumors. However, heterogeneity across CTCs populations and inconsistencies in molecular characteristics between primary tumor cells and CTC present a challenge for clinical applications, which creates a need for a more comprehensive analysis and detailed description of the CTCs^[19]^. We conducted this pilot study of the relative number of CTC subpopulations and a total pool of leukocytes containing ABPs in HNSCC patients. ABPs, which are functional partners in the implementation of actin cytoskeleton remodeling, were selected for the analysis (STRING databases, https://string-db.org). Based on the analysis of the number of subpopulations expressing CFL1, PFN1 and CAP1, the characteristics of CTCs pools and peripheral blood leukocyte pools in patients with HNSCC were given in relation to the main clinical and pathological characteristics of the tumor.

The first phase of the current study was to assess the correlations between serum levels of ABPs and the main clinical and morphological characteristics in patients with HNSCC. Firstly, we found that the CFL1 level was the highest among the studied proteins of the cytoskeleton, and its median level was 0.47 ng/mL (range 0.37–0.82). Secondly, an analysis of the correlations between the studied serum ABPs and the main clinical and pathological parameters revealed that in HNSCC patients with lymph node metastasis (T2–4N1–2M0), the level of PFN1 was almost two times higher than that in HNSCC patients without regional metastases (T1–3N0M0). Thirdly, the ROC analysis determined this indicator as a good classifier of lymph node metastasis (sensitivity, 77%; specificity, 70%). In general, the results obtained are consistent with the existing literature^[4,18]^.

The second phase of the current study consisted of the quantitative analysis of pools of CD45^+^ leukocytes and CD326^+^ CTCs in various groups of patients with HNSCC and counting the subpopulations of these pools containing the studied ABPs. We found that in the peripheral blood of patients with HNSCC, the total pool of CD45^+^ leukocytes mainly consisted of CFL1^+^ and CAP1^+^ subpopulations, and the CD45^−^CD326^+^ CTCs population was most represented by the CAP1^+^ subpopulation. Whereas, in patients with HNSCC without regional metastases, the CFL1^+^CD326^+^ CTCs and PFN1^+^CD326^+^ CTC subpopulations were completely absent, while they appeared in patients with lymph node metastases.

In addition, we found that in HNSCC patients without lymph node metastases, the CAP1^+^CD326^+^ CTC subpopulation was determined, although the labeled CD326^+^ CTCs were not detected in this group. At this stage, it is still difficult to explain these results obtained. This perhaps indicates the establishment of an immunological synapse between the CTCs and immune cells to evade immune responses, or perhaps they were migrating dendritic cells. This is yet to be explored and requires further attention. However, previous research has shown, in oral mucosa through mouse modeling, that the migrating dendritic cells can express CD326 (EpCAM)^[24]^. However, these data require additional research and evidence using cellular technologies.

Analysis regarding the type of epithelium revealed that in HNSCC patients with keratinizing squamous cell carcinoma, the CTC subpopulation expressing CAP1 was 26% lower, compared with that in the patients with non-keratinizing squamous cell carcinoma. Our results are consistent with the existing evidence. The participation of this protein in the processes of tumor differentiation was shown earlier. For example, it has been shown that CAP1 expression was associated with the histological grade and Ki-67 expression in breast cancer^[25]^, and that a high serum level of CAP1 in patients with NSCLC was associated with low tumor differentiation^[9]^.

We then assessed the correlations between the main clinical and morphological parameters of HNSCC patients and the number of subpopulations of CTCs and leukocytes containing the studied proteins. Numerous correlations of different directions and strengths between the studied parameters were revealed, which may indirectly indicate the formation of an immune synapse during tumor growth. The formation and maintenance of the structure of the immune synapse is closely related to the rearrangement of the actin cytoskeleton, the specific mechanisms of which are still poorly understood^[26–27]^.

CFL1 is considered as a biomarker of tumor cell migration and invasion in many tumor diseases^[28]^, and is proposed as a target for antitumor therapy^[29]^. In the current study, significant changes in the number of CTCs expressing CFL1 were shown in patients with HNSCC, depending on the presence of lymph node metastases. The presence of CTCs is known to be a significant marker of cancer metastasis and can be used as a liquid biopsy^[30]^. However, the detection of CTCs in whole blood samples has its nuances and methodological difficulties^[20,31]^. In addition, data are gradually accumulating on the tandem of three ABPs: CFL1, PFN1, and CAP1, the specific mechanism of which during HNSCC progression has not been studied yet. The results obtained indirectly suggest the functional relationship between these proteins. In addition, it can be argued that the study of the molecular characteristics of CTCs, including the dynamics of the actin cytoskeleton, is likely to be useful for understanding the mechanisms underlying tumor progression.

When analyzing the third of the functional partners of ABP-PFN1, it was found that its content in the serum was almost two times higher in HNSCC patients with lymph node metastasis (T2–4N1–2M0) than that in patients without metastases (T1–3N0M0). The ROC analysis determined that the following parameters could be the most valuable classifiers: the serum levels of CAP1 (sensitivity, 84%, specificity, 70%) and PFN1 (sensitivity, 77%, specificity, 70%) as well as the number of PFN1^+^CD326^+^ CTC subpopulations. Assessment of the number of PFN1^+^CD326^+^ CTCs may be the most valuable indicator for the risk of lymph node metastasis: AUC, 0.92 (95% CI, 0.80–1.00), sensitivity, 85%, and specificity, 96%. The analysis of survival showed that the serum level of PFN1 could be used to predict the one-year metastasis-free survival of HNSCC patients. When evaluating the diagnostic parameters, it was determined that the optimal threshold value (cut-off point) for the serum level of PFN1 was 0.32 ng/mL. Within a 12-month follow-up, lymph node metastases were detected in 64.7% (11 patients out of 17) of HNSCC patients with a PFN1 level of more than 0.32 ng/mL before treatment. Thus, the baseline serum PFN1 level of 0.32 ng/mL can be taken as a critical point for the formation of risk groups for predicting a poor outcome in patients with HNSCH. Considering the small sample of HNSCC patients and the *P*-value of 0.07, the results of this analysis require validation, although the results obtained in our study are consistent with the literature data^[4]^.

The populations of CD45^+^ leukocytes and CD326^+^ CTCs were mostly represented by subpopulations expressing CAP1; however, the results did not reach the threshold for statistical significance. Significant differences were obtained only in HNSCC patients with different degrees of epithelial keratinization, which is generally consistent with the data on the involvement of CAP1 in the differentiation of certain cell types, in particular, neurons and lemmocytes^[32]^.

The actin cytoskeleton plays a very important role in many processes during metastasis^[33–34]^, including not only cellular locomotion, but also mitosis, cytokinesis, intracellular transport, endocytosis, exocytosis, and secretion. Actin reorganization is regulated by a large group of ABPs and is currently being actively studied for the diagnosis and prognosis of various diseases, including cancer^[28,32,35]^.

### Conclusion

The current study provides a new perspective on a number of ABP-expressing CTCs and peripheral blood immune cells in HNSCC patients. Considering that the studied ABPs are functional partners, differences in levels of these proteins in CTCs and blood leukocytes are of fundamental interest. The increase in the number of PFN1-expressing CTCS in HNSCC patients with lymph node metastases and the dependence of the number of CAP1-containing CTCs on the histotype of the primary tumor may be associated with the acquisition of functional features of CTCs during the progression of HNSCC. Correlations between the number of CTCs and immune cells expressing CAP1 and PFN1 may reflect the peculiarities of the molecular mechanisms of the tumor-host relationship in the pathology. Targeted manipulation of ABP expression both in the tumor and in the CTCs is likely to be used to suppress the proliferation and migration of tumor cells. At least, the dependence of the content of PFN1^+^CD326^+^ CTCs on lymph node metastasis could become a prerequisite for the development of new additional criteria for determining the risk of metastasis in HNSCC patients. The results obtained here indicate the need to study this phenomenon to decide whether this parameter can be used as an additional prognostic criterion for the risk of tumor progression (lymph node metastases).

## Fundings

None.
